# *eLogUp!* A precise, affordable and open-source IoT data logger to scale-up long-term environmental monitoring

**DOI:** 10.1016/j.ohx.2025.e00660

**Published:** 2025-05-29

**Authors:** Franck Perret, Ilane Cherif, Frédéric Cherqui, Nicolas Walcker, Adrien Barra, Bastien Bourjaillat, Laëtitia Bacot, Oldrich Navratil

**Affiliations:** a UMR 5600 Environnement, Ville ET Société, Université de Lyon, CNRS, Lyon, France; b Université de Lyon, INSA Lyon, DEEP, UR7429, 69621 Villeurbanne, France; c School of Agriculture, Food and Ecosystem Sciences, The University of Melbourne, Burnley, VIC 3121, Australia; d GRAIE – 66 Boulevard Niels Bohr – CS 52132, 69100 Villeurbanne Cedex, France

**Keywords:** Arduino MKR, Field monitoring, River monitoring, Low-cost, Low-tech, LoRaWAN, Sensors, Environmental time series

## Abstract

eLogUp! is a low-cost, modular, and open-source data logger designed to support both research and operational environmental monitoring applications requiring accurate data recording and storage, efficient power management, and reliable data transmission. It serves as a key component for the implementation of effective Low-Cost Network Sensor (LCNS) systems, enhancing environmental observation capabilities. eLogUp! consists of a custom-designed PCB integrated with an Arduino® MKR microcontroller, enabling data acquisition from a wide range of analog and digital sensors, such as temperature, water level, and humidity probes, with user-defined sampling intervals. A distinctive feature of eLogUp! is its auto-wake-up function, which powers down the system between measurements to optimize energy consumption. It also integrates several electronic components (e.g. RTC) to minimize the overall footprint of the system. The system can thus operate on a small LiPo battery and solar panel. Data transmission can be carried out using different protocols (e.g., WiFi, GSM, or LoRaWAN). Developed as an alternative to commercial environmental data loggers, which are often expensive, overly specialized, proprietary, or limited to a narrow set of parameters, eLogUp! offers an affordable, adaptable, and open-source solution. This makes it a versatile tool for environmental researchers, field practitioners, and citizen science initiatives, providing a scalable and accessible approach to long-term environmental data collection.

## Graphical abstract

**Figure:**
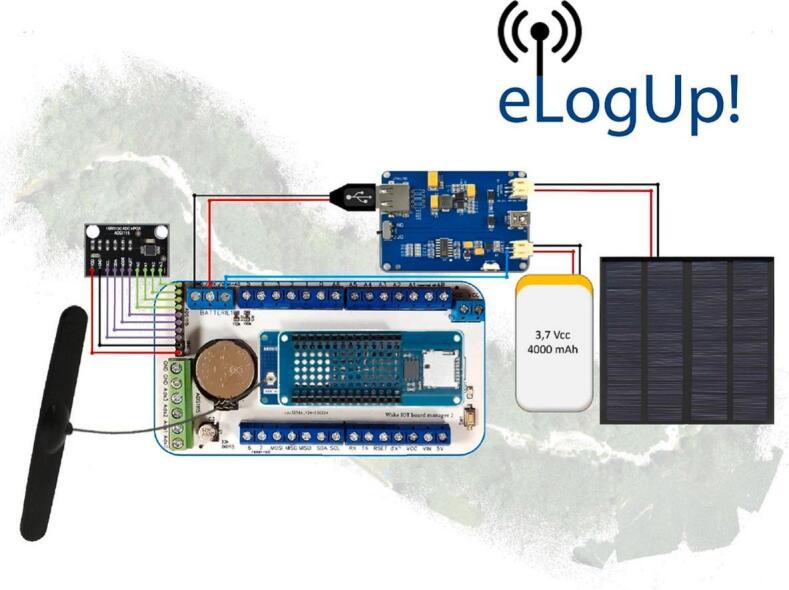

Specifications table.

**Table:** 

Hardware name	eLogUp!
Subject area	• *Engineering and materials science* • *Educational tools and open-source alternatives to existing infrastructure*
Hardware type	• *Field measurements and sensors* • *Mechanical engineering and materials science*
Closest commercial analog	• Campbell Datalogger CR1000X ∼ 1500€ • Milesight datalogger ∼ 150€
Open-source license	GNU General Public License (GPL) 3.0
Cost of hardware	∼230€
Source file repository	https://osf.io/96zv2/?view_only=5a670b5f49634e0cb3b69654044ec012

## 1 Hardware in context

In environmental sciences, data loggers are essential electronic devices used to measure and record environmental parameters over time. They automatically transmit the collected data, enabling the construction of comprehensive long-term time series for analysis [1]. Data loggers capture signals from a variety of sensors (e.g., temperature, rainfall, humidity, water levels, air quality) and store the collected measurements for further analysis [2]. In some applications, data loggers can also trigger actions, such as automatic sampling (e.g., water or air) or pictures, based on specific measurement thresholds (e.g., water turbidity or level). Depending on the model and application, data loggers may transmit data remotely to web platforms.

The emergence of IoT-based environmental monitoring systems has greatly advanced environmental monitoring capabilities [3]. These IoT systems facilitate spatially distributed monitoring across numerous locations, including remote areas, enabling a deeper understanding and more effective tracking of the 'hot moments' and 'hot spots' of biophysical and geochemical processes within ecosystems [4]. Remote data transmission supports efficient maintenance of monitoring stations by allowing managers to respond promptly to anomalies in the data (e.g., sensor failure, low battery levels, or vandalism). This capability for remote pre-diagnosis of the monitoring network ensures high-quality data acquisition with minimal gaps in time series records. It is particularly crucial for maintaining system operability during rare and intense events, such as thunderstorms, floods, or pollution incidents.

Connecting environmental datalogger to a web platform is thus essential for developing dense sensor networks, particularly when sensors are deployed in challenging or remote environments (e.g., high-altitude mountains with cold and moisture conditions, rivers during floods, in caves or wastewater network). Given their field deployment, IoT-based environmental dataloggers must also be designed for minimal and highly optimized energy consumption to limit the need for field interventions, relying on power sources such as batteries or solar panels. Additionally, they must be discreet and robust, capable of withstanding extreme environmental conditions (e.g., extreme temperatures, high humidity, or urban areas subject to degradation).

Commercial data loggers have been available for many years and are recognized for their reliability and robustness (e.g., Campbell Scientific, OTT). However, their high cost (>1500 euros) limits their deployment across multiple locations[13]. Some of these systems offer IoT functionalities, either integrated into the data logger, or as additional components in their measurement stations (e.g., Vega, OTT, National Instruments, Endress, Hach, or Nivus). More recently, with the aim of supporting dense sensor networks, companies have introduced more affordable solutions tailored to monitoring specific environmental parameters, such as CO2 concentration, humidity, and water levels. These systems generally provide at least one method of remote data transmission via LoRaWAN or SigFOX networks (e.g., Milesight, Atim, Tektelic, Adeunis, or Dragino).

However, these solutions often lack modularity and fail to offer the open-source flexibility required for user modifications or repairs. In extreme cases, some systems have non-replaceable batteries (e.g., Diver® or Hobo®), rendering them obsolete after a few years of operation. Additionally, many remote data transmission systems do not include local data storage (e.g., via SD cards), leading to data loss if the communication network fails. Moreover, precise time stamping is not commonly prioritized, as it may not be necessary for certain applications (e.g., alert). For research purposes, however, accurate time stamping is crucial for analyzing and comparing time series data across multiple monitoring stations, enabling causal inference analysis [5].

In recent years, numerous research projects have proposed low-cost, Arduino-based dataloggers as viable alternatives to traditional commercial systems [2,[6], [7], [8], [9], [10], [11], [12], [13], [14]]. All these initiatives aim to reduce costs for scientific or operational purposes while reclaiming control over measurement technologies [3,15,16,17]. These low-cost and open-source data loggers serve as the foundation for Low-Cost Sensor Networks (LCSN) designed for monitoring air pollution or urban water systems [3]. However, few low-cost IoT data loggers currently integrate multiple essential features into a single system [2], such as the ability to connect multiple sensors (digital or analog), to secure data recording on an SD card with a precise timestamp, and to provide a low-tech yet weatherproof enclosure. Furthermore, the paradigm shift introduced by LCSN highlights the deployment of low-cost IoT sensors across numerous field locations, often in remote areas. Achieving this goal is feasible only if the data logger supports data transmission to a web server and operates with extremely low energy consumption [6]. To address these requirements, such systems should leverage low-cost, open-source data transmission protocols, such as LoRaWAN. Furthermore, they must incorporate ultra-low energy consumption, autonomous power supply capabilities, and integrated power consumption monitoring to enable efficient system oversight and prompt operator intervention in the event of malfunctions.

In this study, we present eLogUp!, a cost-effective datalogging system priced about 200 euros. This versatile platform is designed to record signals from a diverse array of sensors, encompassing both digital and analog types as well as low-cost and conventional models. eLogUp! supports multiple data transmission methods, including GSM, LoRaWAN, WiFi, and Bluetooth. It was developed with a focus on robustness, open-source accessibility, modularity, and affordability, aiming to minimize hardware costs while maximizing energy efficiency. Key optimizations include: i) minimizing data drift and ensuring precise time stamping, ii) enabling multi-channel recording with enhanced measurement accuracy and simplified sensor integration, iii) achieving low power consumption for extended autonomy through the use of a LiPo battery, regulator, solar panel, and power supply monitoring, and iv) employing a robust, discreet, low-tech, and waterproof encapsulation suitable for field applications. The system's design and performance was i) tested in lab to evaluate and improve batteries performance, and ii) demonstrated through a year-long field survey, measuring air temperature, humidity, and battery levels.

## 2 Hardware description

### 2.1 General description

**Fig. 1:**
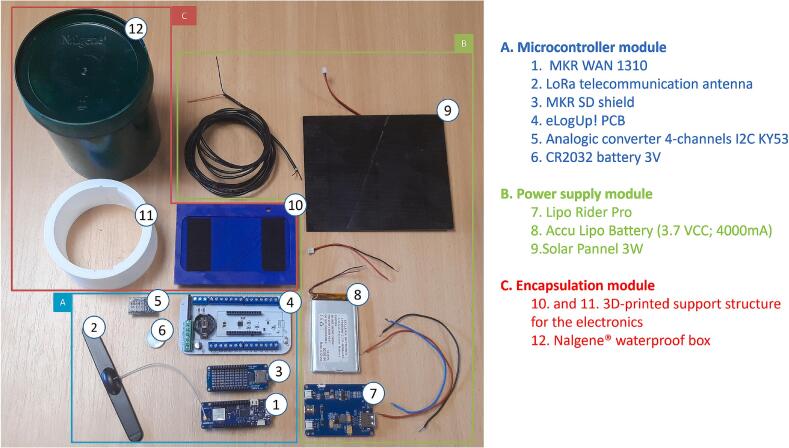
The eLogUp! monitoring system with its three modules: (A) the microcontroller module with the eLogUp! PCB, the MKR microcontroller (here the MKR WAN1310 model), an SD module (MKR MEM SD shield) and an optional analogic converter; (B) the power supply module with the battery, the Lipo card and a solar panel; and (C) the encapsulation materials (a painted Nalgene® waterproof box, cable glands, and a 3D-printed support structure for the electronics).

The eLogUp! monitoring station is organized into three main modules (Fig. 1): i) a microcontroller module (A), ii) a power supply module (B), and iii) the encapsulation (C). Module A includes the eLogUp! PCB (Printed Circuit Board), an Arduino MKR microcontroller, a telecommunication antenna, a SD card data acquisition board, and an optional digital-to-analog signal converter. Module B includes the system's power supply components, such as the battery, solar panel, and regulator for recharging the battery. Module C ensures the protection of the data logger, preserving the longevity of the electronics in harsh environments. It features a simple, discreet, and durable encapsulation, consisting of a Nalgene® waterproof box, cable glands, and a 3D-printed support structure for the electronics. These three modules are described in detail below.

### 2.2 eLogUp! PCB

**Fig. 2:**
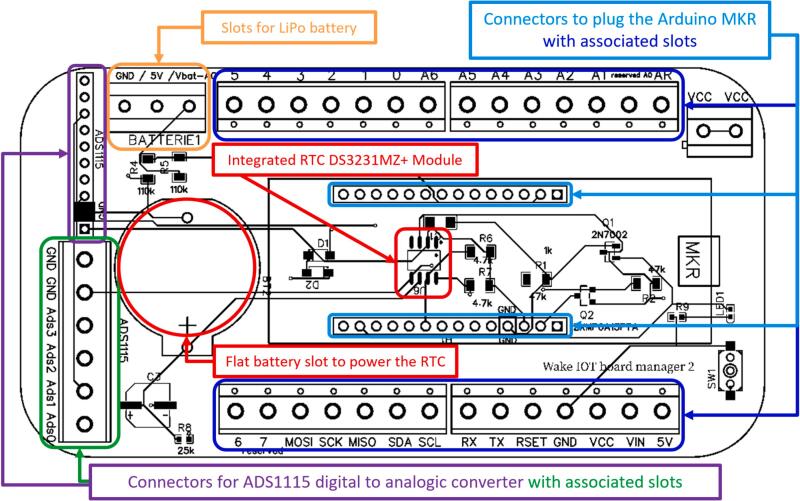
Front view of the eLogUp! PCB, showcasing its key components and connectors, including the terminal blocks for sensor connections, power supply inputs, optional digital-to-analog converter slots, and the DS3231MZ + Real-Time Clock (RTC) module.

The core hardware component of the system is the eLogUp! PCB (Fig. 1A), specifically designed to integrate a variety of low-cost electronic devices. At its center, the PCB features two rows of 14 connectors tailored for Arduino MKR hardware (Fig. 2). These connectors are directly connected to two corresponding rows of 14 terminal blocks on the PCB, providing a secure and reliable interface for connecting sensor wires to the datalogger.

**Fig. 3:**
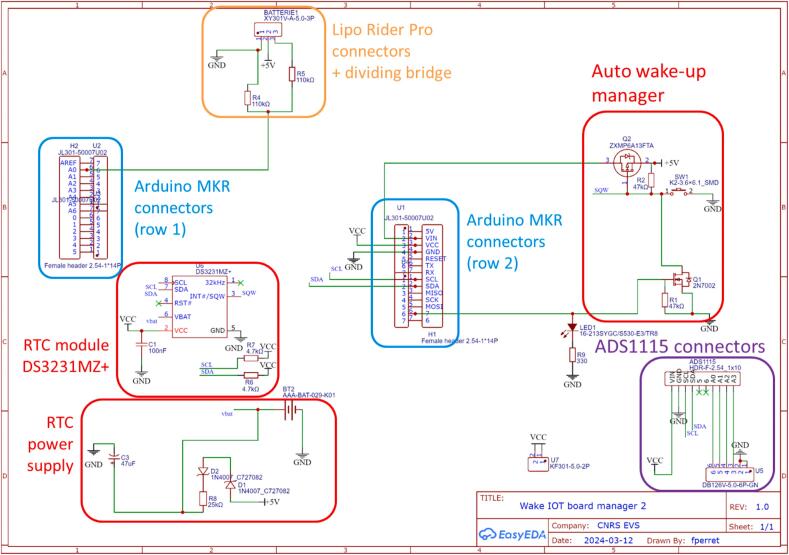
Schematic diagram of the eLogUp! PCB, illustrating the integration of the Wake IoT board manager.

The power supply connectors on the eLogUp! PCB includes three dedicated terminal blocks for power supply connections: VCC+ (+5V), the negative ground (GND-), and Vbat, which allows measurement of battery voltage via pin A0 on the MKR (exclusively reserved for this purpose). The PCB also features a row of 10 connectors to support an optional KY-0053 or ADS1115 [21] digital-to-analog converter (Fig. 1, Fig. 3). This component provides four additional analog inputs (accessible through corresponding terminal blocks) and delivers improved measurement accuracy compared to the default MKR analog pins (A1 to A6; Gisi et al., 2024). Additional VCC + and GND- terminal blocks are available as auxiliary outputs for powering external sensors. Furthermore, the board is equipped with a DS3231MZ + Real-Time Clock (RTC) module [20], which ensures accurate time stamping and supports eLogUp!'s auto-alarm functionality. To maintain timekeeping during power interruptions, the RTC module requires a CR2032 (or CR2016) button cell battery.

### 2.3 Arduino MKR microcontrollers

**Fig. 4:**
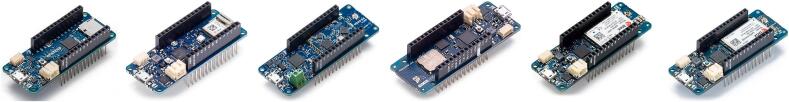
Arduino MKR cards (from left to right): MKR ZERO; MRK WiFi 1010; MKR FOX 1200; MKR WAN 1310; MKR GSM 1400; MKR NB 1500.

In this paper, we present almost exclusively the results obtained using the MKR WAN 1310 board. This board offers interesting prospects in the field of IoT as it enables connection to the LoRaWAN network. However, many other boards exist in the Arduino MKR series [18], all of which are compatible with the eLogUp! PCB (Fig. 4). Each of them is designed to meet specific measurement requirements based on the project needs and field constraints, such as cost, the GSM or LoRaWAN network availability or necessity (Table 1).

**Table 1:** Tested MKRs and feedback on their use. This paper mainly focuses on the use of the MKR WAN 1310 model.

Model	Compatibility with *eLogUp!* PCB	Communication network	Signal’s strength	Range of detection	Feedback	Price
MKR ZERO	Complete	/	/	/	Simple and cost-effective for applications without wireless communication. The onboard SD port is particularly useful for data logging without additional accessories.	31,57 €
MKR WiFi 1000/1010	Supposedly, but not tested	WiFi	10 mW −100 Mw	∼500 *m*	For applications requiring internet connectivity. The onboard WiFi module performs reliably in urban environments but may struggle with range in rural areas.	40,22 €
MKR FOX 1200	Complete	Sigfox	1 mW −10 mW	∼15 km	Highly efficient for long-range communication in rural or remote areas.	Unavailable
MKR WAN 1300/1310	Complete	LoRaWAN	1 mW −10 mW	∼15 km	Highly efficient for long-range communication in rural or remote areas. The low power consumption makes it suitable for battery-powered systems, though range and performance can vary with environmental conditions.	49,50 €
MKR GSM 1400	Supposedly, but not tested	GSM	10 mW −100 mW	∼50 km	Suitable for remote areas lacking WiFi or LoRaWAN infrastructure. It provides reliable communication over cellular networks but consumes more power compared to other models.	Unavailable
MKR NB 1500	Complete	NB-IoT	1 mW −100 mW	∼50 km	Ideal for low-power, wide-area applications. NB-IoT coverage, i.e. the low frequencies of the GSM telephone network (NB for Narrow Band), may be limited in some regions, which affects the board's utility in certain locations.	96,40 €

The MKR ZERO stands out as the only model with an onboard micro SD port, removing the need for an additional MEM Shield SD. Other MKR boards offer enhanced capabilities, such as support for remote communication via multiple protocols. This versatility makes them well-suited for applications that require wireless connectivity or long-distance data transmission, ensuring adaptability across diverse use cases.

Among the boards listed in Table 1, the MKR WiFi 1010 is the least suitable for field applications due to its limited detection range and high power consumption for telecommunication. As a result, microcontroller selection often focuses on LP-WAN (Low-Power Wide-Area Network) protocols like LoRaWAN and Sigfox, which offer low energy consumption and long-range communication. These networks are especially well-suited for use with low-cost sensors. However, the MKR FOX 1200 is no longer commercially available, and access to the Sigfox network requires an annual subscription. In contrast, LoRa networks are free to use within private or community-based infrastructures [22]. It supports bidirectional communication with antennas and relays located several hundred meters to kilometers away in obstacle-free environments. Therefore, the MKR WAN 1310 is our recommended choice for IoT applications. However, this solution relies on the availability of community-installed antennas or it requires a subscription to a private network operator.

The MKR 1400 and MKR 1500 utilize GSM (Global System for Mobile Communications) networks, similar to those used by cell phones. These boards can connect to 2G (which is declining), 3G, or 4G networks using a standard SIM card. GSM-based communication simplifies data transfer by leveraging the SIM card's data plan and supports sending and receiving SMS messages, enabling remote interaction with the measurement device, such as sending commands or receiving alerts. The MKR 1500 is particularly advantageous due to its lower energy consumption, thanks to support for NB-IoT (Narrow Band IoT), which operates using low-power radio waves. This makes it an excellent choice for remote applications. Currently, we are focusing on deploying the MKR NARROWBAND 1500, as its extensive cellular network coverage makes it ideal for remote measurement systems, particularly in regions where the LoRa network is either limited or unavailable. Additionally, both GSM and WiFi communication enable over-the-air (OTA) programming, allowing Arduino code to be updated remotely. This feature is especially useful during the initial deployment phase of field systems, facilitating easier maintenance and updates without requiring physical access to the devices. It is worth mentioning that GSM (2G, 3G or more) or WiFi communications lead to higher energy consumption compared to low-power communication such as SigFox, LoRaWAN or NB-IoT). GSM and WiFi consumme more energy during data transmission (compared to low-power communication). Moreover, if the system needs to receive instructions over the air, it must stay connected to the network.

### 2.4 MKR MEM shield SD

**Fig. 5:**
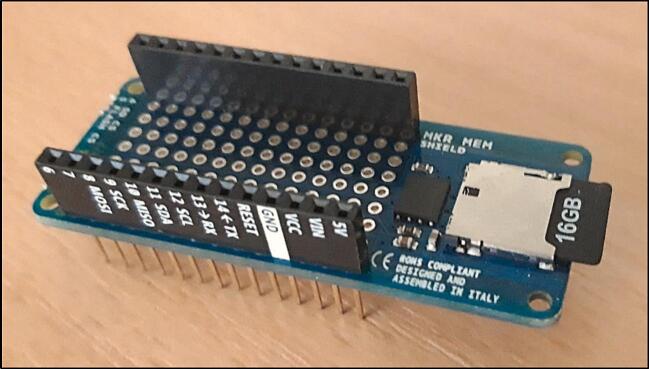
MKR MEM Shield SD, with a 16 Gb SD Card.

The MEM Shield SD card for MKR (Fig. 5) enables data storage on a standard SD card, with a recommended capacity of 16 GB. The SD card contains at least one text file where the measurement logs (datalogs) are stored. The microcontroller handles the incremental addition of new data to this file. The datalog format is similar to a.CSV file, using the semicolon (“;”) as the delimiter. Each line in the file corresponds to a measurement timestep, ensuring an organized and easily exportable data structure.

### 2.5 Power supply

**Fig. 6:**
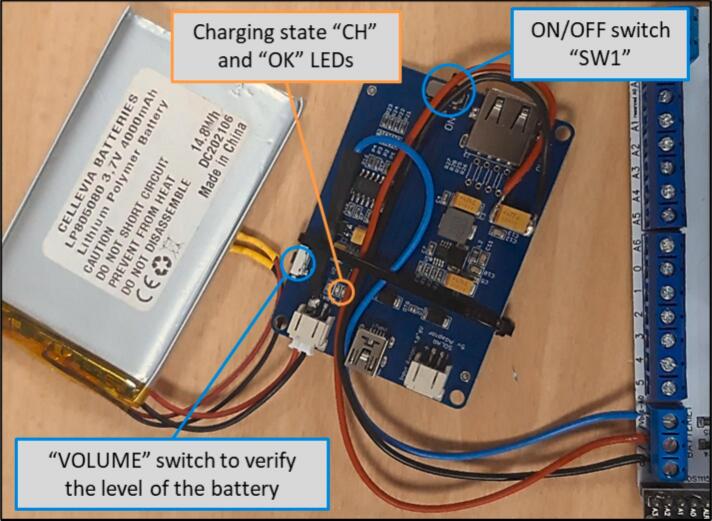
Connecting the Lipo battery to the LiPo Rider Pro regulator and the eLogUp! PCB.

The eLogUp! system relies on a LiPo battery to power the various devices connected to the PCB (Fig. 1, Fig. 2). To enable battery recharging during operation, the system incorporates a LiPo Rider Pro module, which facilitates charging via a solar panel. In remote or inaccessible deployment locations, monitoring the battery voltage is essential. Therefore, the “Vbat” terminal block on the eLogUp! PCB directly receives the battery's voltage (through the blue wire, as shown in Fig. 6) before it is processed by the regulator.

The solar panel significantly extends the battery's lifespan by providing efficient, continuous recharging, depending on sunlight availability. Furthermore, the eLogUp! PCB reduces energy consumption by enabling the system to be powered on and off as required. This is accomplished through the control of a transistor (Q1, Fig. 2), which is activated or deactivated by the DS3231MZ + RTC module when an alarm is triggered via Arduino code (Design File 2). Unlike the “sleep” and “deepSleep” functions provided by the Arduino Low Power library, this approach allows the board to be completely powered down between measurement cycles. Consequently, energy consumption between measurements is negligible, ensuring optimal efficiency for long-term, autonomous operation.

### 2.6 3D printed casing and encapsulation

**Fig. 7:**
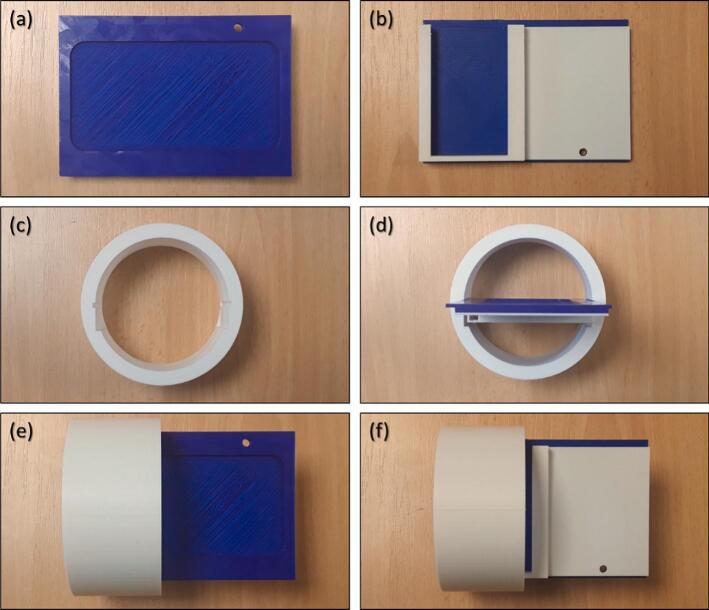
3D printed card support. Plate: front (a), back (b). (c) Cylinder only. (d) Cylinder and plate: recto (e), verso (f).

The system encapsulation is simple yet effective, utilizing a Nalgene® box to shield the electronics from moisture, dust, and animals (e.g., ants; Fig. 1). The box is modified with two or three holes, depending on the number of sensors, to accommodate IP65-rated watertight cable glands for the sensor and solar panel wires. To further protect the electronics from humidity, several desiccant gel sachets can be placed inside the box. The electronics are securely mounted within the box using a 3D-printed bracket (Fig. 7). This bracket consists of a plate designed to hold the *eLogUp!* PCB at the front and the LiPo Rider Pro module with its battery at the back, as well as a cylinder with notches to stabilize the plate within the Nalgene® box. This design ensures easy access to key components, including the “SW1” interrupt button on the *eLogUp!* PCB, the SD card, and the ON/OFF switch on the LiPo Rider Pro module. The necessary files for 3D printing these components are provided in Design File 3.

## 3 Design files summary

**Table 2:** Design Files in the repository.

**Design File name**	**File type**	**Open source license**	**Location of the file**
Design File 1	.zip	GNU General Public License (GPL) 3.0	In the repository
Design File 2	.zip file (with.ino files inside)	GNU General Public License (GPL) 3.0	In the repository
Design File 3	.stl	GNU General Public License (GPL) 3.0	In the repository
Design File 4	.avi	GNU General Public License (GPL) 3.0	In the repository

Design File 1 is the.zip folder containing every document needed to manufacture the PCB: Schematics, PCB, Gerber files, 3D model….

Design File 2 contains Arduino files with the basic “program skeleton” depending on the MKR used: Code_*eLogUp*_LoRaWAN and Code_*eLogUp*_NB-IoT (Arduino IDE is needed to compile and upload the program into the MKR).

Design File 3 is the.stl file to 3D print the casing.

Design File 4 is a video tutorial providing all the visual instruction to assemble any of the cheap’eau system and turn on the MKR using the PCB. + shut down the system properly.

## 4 Bill of materials

To manufacture the eLogUp! PCB through JLCPCB.com or similar platforms, only the Gerber, BOM, and CPL files found in the “Design File 1” folder are required (refer to the list of design files in Table 2).

**Table 3:** Bill of materials (prices at 03/18/2025).

**Designator**	**Component**	**Number**	**Cost per unit −Euros**	**Total Cost −Euros**	**Source of materials**	**Material type**
A1	Printed Circuit Board with components	1	20 €	20 €	JLC PCB: https://jlcpcb.com	Other
A2	Battery 3 V CR2032 (ou CR2016)	1	1.14 €	1.14 €	RS, Article code: 186-3376 https://fr.rs-online.com/web/p/piles-boutons/1863376?gb= s	Lithium-manganèse
A3-1	Arduino MKR WAN 1310 microcontroller Other MKR: ZERO, or 1000, or 1010, or 1200, or 1400, or 1500	1	49.5 € From ∼ 30 € to ∼ 90 €	49.5 € From ∼ 30 € to ∼ 90 €	Go Tronic, Article code: 36,501 https://www.gotronic.fr/art-carte-arduino-mkr-wan-1310-abx00029-30753.htm	Other
A3-2	LoRa telecommunication antenna	1	5.90 €	5.90 €	Go Tronic, Article code: 35,640 https://www.gotronic.fr/art-antenne-sigfox-lora-et-gsm-x000016-27327.htm	Other
A3-3	Universal SIM card with 500 MB data volume and 250 SMS (Only for MKR 1400 or 1500)	1	10 €	10 €	1nce® https://1nce.com/en-eu/resources/downloads/1nce-infographic-download	Other
A4-1	Module SD Arduino MKR MEM Shield	1	24.40 €	24.40 €	Go Tronic, Article code: 36,137 https://www.gotronic.fr/art-shield-mkr-mem-asx00008-28737.htm	Other
A4-2	MicroSD card 16 GB	1	8.74 €	8.74 €	RS, Article code: 180-5793 https://fr.rs-online.com/web/p/cartes-sd/1805793	Other
A5 (modulable)	Analog converter 4 channels I2C KY053	1	9,90 €	9,90 €	Go Tronic, Article code: 35,746 https://www.gotronic.fr/art-convertisseur-analogique-4-canaux-ky053-27692.htm	Other
B1-1	Accu LiPo 3.7 Vcc 4000 mAh	1	23.50 €	23.50 €	Go Tronic, Article code: 09,948 https://www.gotronic.fr/art-accu-lipo-3–7-vcc-4000-mah-l805080-31843.htm	Lithium polymer
B1-2	JST connector to plug the battery to the LiPo Rider Pro	1	0.40 €	0.40 €	Go Tronic, Article code: 48,914 https://www.gotronic.fr/art-cordon-a-connecteur-jst-2-contacts-22583.htm	Other
B2-1	LiPo Rider Pro card	1	15.60 €	15.60 €	Go Tronic, Article code: 31,355 https://www.gotronic.fr/art-carte-lipo-rider-pro-106990008–19050.htm	Other
B2-2	USB A − micro-USB A M/M cable	2	1.95 €	3.90 €	Go Tronic, Article code: 48,314 https://www.gotronic.fr/art-cordon-50-cm-rs105-33657.htm	Other
B2-3	Wire to be soldered to the LiPo Rider Pro	∼5cm	0.87 €/meter	∼0.05 €	RS, Article code: 201-2709 https://fr.rs-online.com/web/p/fils-de-cablage/2012709	Other
B3-1	Solar Cell SOL3W	1	19 €	19 €	Go Tronic, Article code: 27,134 https://www.gotronic.fr/art-cellule-solaire-sol3w-18996.htm	Silicium
B3-2	Waterproof extension for the red and black wires of the solar cell	1	9.58 €	9.58 €	RS, Article code: 253-7022 https://fr.rs-online.com/web/p/cables-d-alimentation/2537022	Other
C1	Nalgene® 1L jar to protect every element	1	16.5 €	16.5 €	Nalgene https://nalgene.com/product/32oz-nalgene-storage-jar/	Tritan™
C2	Pack of 10 waterproof cable glands for 5 to 10 mm cable	2 or 3 out of 10	16.75 €	5.03 €	RS, Article code: 822-9653 https://fr.rs-online.com/web/p/presse-etoupes/8229653	Nylon PA66
C3	3D printed case to lock every element (PET-G filament)	130 g	36.91 €/kg	5.75 €	RS, Article code: 190-1959 https://fr.rs-online.com/web/p/materiaux-pour-impression-3d/1901959	Polyéthylène Téréphtalate
TOTAL (based on an MKR WAN 1310)	/	/	/	228.89 €	/	/

**Table 4:** Battery tests in lab with different configurations (without solar panel).

Test	Number of days of functioning	Number of measurements	Type of « wait » mode	Description
a	3	270	delay	MKR powered by using the LiPo Rider Pro regulator
b	4	385	sleep	
c	5	403	deepSleep	
d	23	2 103	deepSleep	MKR powered through JST port
e	35	3 386	Auto wake-up	Normal use of the *eLogUp!* PCB: the MKR is powered through the VIN pin **with** the LiPo Rider Pro, and turns off between each measurement.
f	97	9 198	Auto wake-up	The same than (e) but **without** the LiPo Rider Pro

## 5 Build instructions

### 5.1 Assembling eLogUp! system

**Fig. 8:**
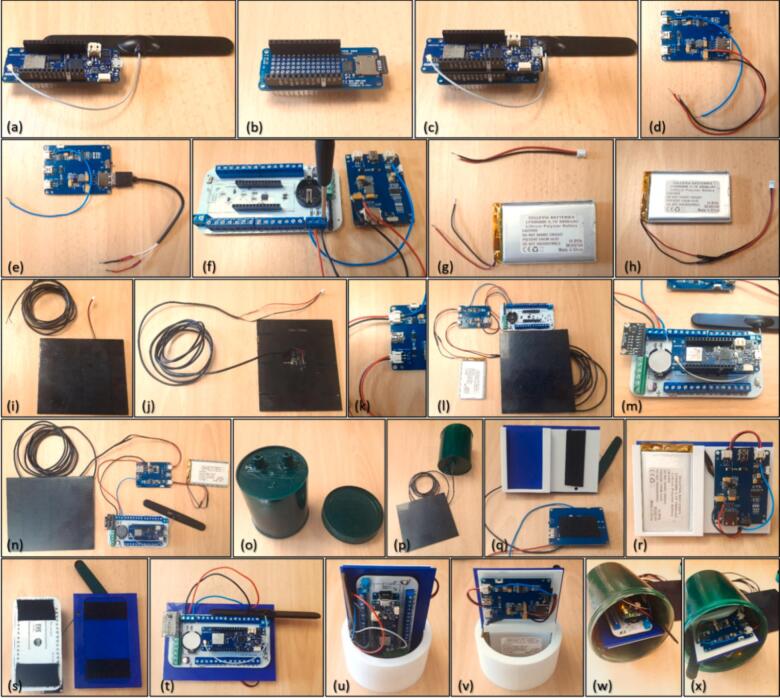
Assembly steps of the eLogUp! system, featuring the 3D-printed support and cost-effective encapsulation.

The assembly process for the eLogUp! system (Fig. 8) is thoroughly documented in the video tutorial available in Design File 4, with the key steps summarized below. All items (e.g., A3-1) are listed and described in Fig. 1 and Table 3.

a) Connect the telecommunications antenna to the MKR board and insert the SIM card, if required (items A3-1, A3-2, and A3-3, depending on the model).

b) Insert the SD card into the MKR MEM SD Shield (items A4-1 and A4-2).

c) Place the selected MKR board onto the MEM Shield SD, aligning the pins according to their labels (items A3-1 and A4-1).

d) Option 1: Solder two 10 cm wires to the C13 capacitor terminals on the LiPo Rider Pro board (red for “+”, black for “-”) (items B2-1 and B2-3).

e) Option 2: Strip a USB cable to access the power leads (red for “+”, black for “-”; ensure with a voltmeter that the polarities are not reversed.) and connect the USB plug to the LiPo Rider Pro (items B2-1 and B2-2).

f) Connect the three wires from the LiPo Rider Pro to the GND, 5 V, and Vbat terminals on the eLogUp! PCB (items B2-1 and B2-3).

g) Cut the wires from the LiPo battery one at a time to prevent any shortcut and replace the output port with a JST connector.

h) Identify with a voltmeter the positive and negative terminals of the battery and match them with the LiPo Rider Pro’s polarity (items B1-1 and B1-2). Ensure no short circuits occur.

i) and j) Extend the solar panel cable to the desired length using a watertight extension if necessary (items B3-1 and B3-2).

k) and l) Ensure the LiPo Rider Pro switch is set to OFF, then connect the LiPo battery and solar panel to their respective ports (items B2-1 and B2-3).

m) Attach the following components to the eLogUp! PCB (item A1): a CR2032 or CR2016 battery in its slot (item A2); the MKR board with MEM Shield SD (items A3 and A4); an analog-to-digital converter, if used (item A5).

n) At this stage, the datalogger is operational, and sensors can be connected to the terminal blocks linked to the microcontroller.

o) Drill two or more holes in the bottom of the Nalgene® box to insert IP65-rated cable glands for the solar panel and sensor cables (items C1 and C2). Secure these glands with glue, and paint the box for camouflage, if necessary.

p) Insert the solar panel cable through one of the cable glands.

q) Print the 3D bracket components (Design File 3) using the desired material.

r) Insert the battery into its case and attach the regulator using adhesive tape or Velcro.

s) Secure the eLogUp! PCB to the opposite side of the bracket.

t) Pass the three LiPo Rider Pro wires through the dedicated hole in the bracket (item C3).

u) and v) Insert the bracket into the notches of the 3D-printed cylinder, ensuring the system remains stable within the Nalgene® box. Add a desiccant sachet for humidity protection.

w) and x) Before sealing the box, connect the solar panel to the regulator and test the system by pressing the SW1 button on the eLogUp! PCB. Ensure the SW1 button, SD card slot, and LiPo Rider Pro ON/OFF switch are easily accessible from the top of the Nalgene® box.

At the end of step (n), the system's wiring should match the diagram in Fig. 3 (although the microcontroller may differ from the MKR WAN 1310). For complete protection, use the recommended Nalgene® box (Table 3) or an equivalent waterproof enclosure, following steps (o) to (x).

## 6 Operation instructions

### 6.1 Launching the datalogger and program execution

**Fig. 9:**
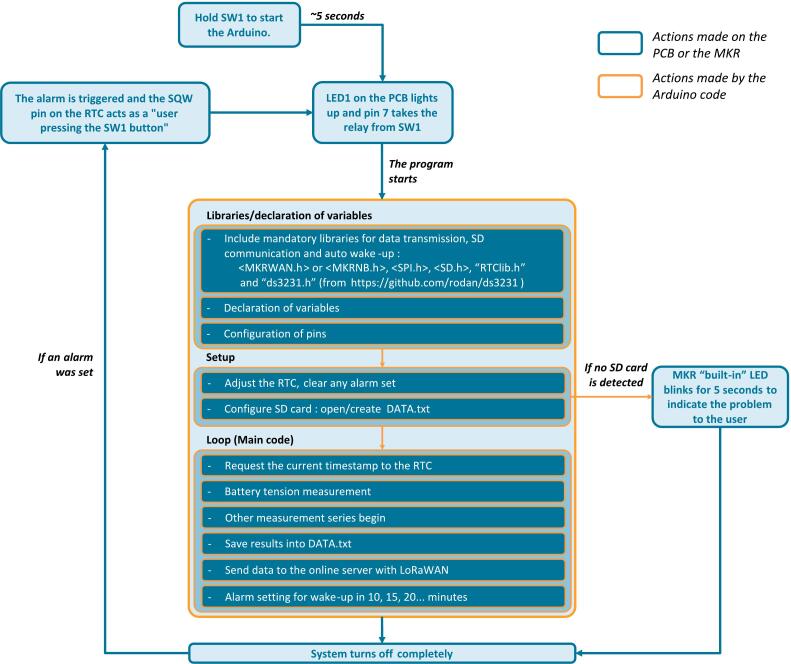
System activation and execution of the eLogUp! program (Arduino script in Design File 3).

An Arduino code is a program written in C++ within the Arduino Integrated Development Environment (IDE). It consists of two main functions: “setup()”, which is executed once when the board is powered on, and “loop()”, which runs continuously until the board is powered off. Typically, the code begins by including necessary libraries and declaring global variables required for the program. However, for scripts specifically tailored to the operation of the eLogUp! system (Fig. 9), the board is programmed to shut down immediately after completing a single loop cycle. This approach ensures minimal power consumption between measurements, optimizing the system for low-energy operation.

**Fig. 10:**
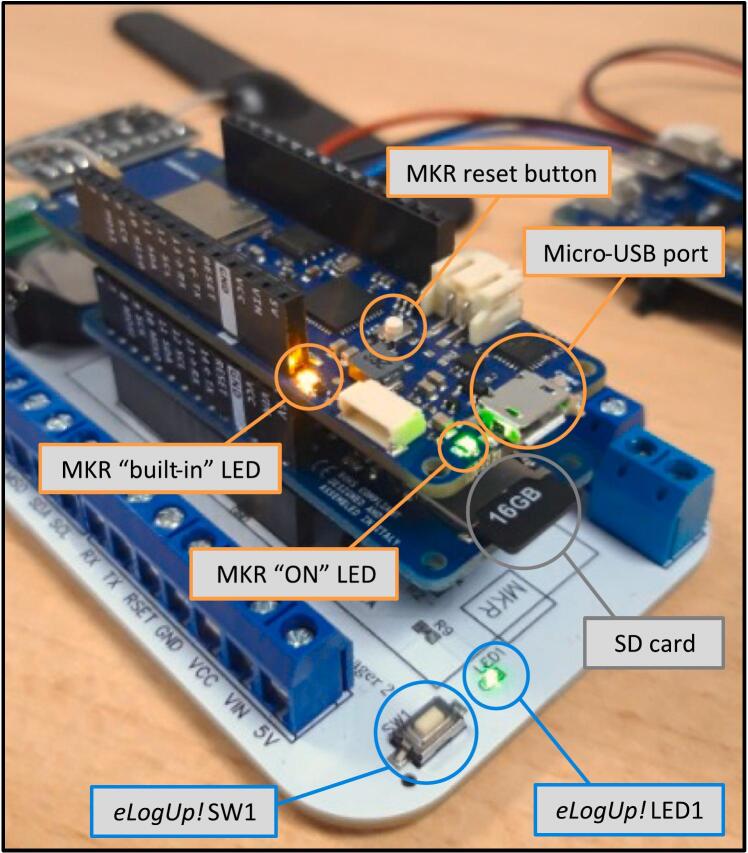
Key components for optimal datalogger operation.

**Fig. 11:**
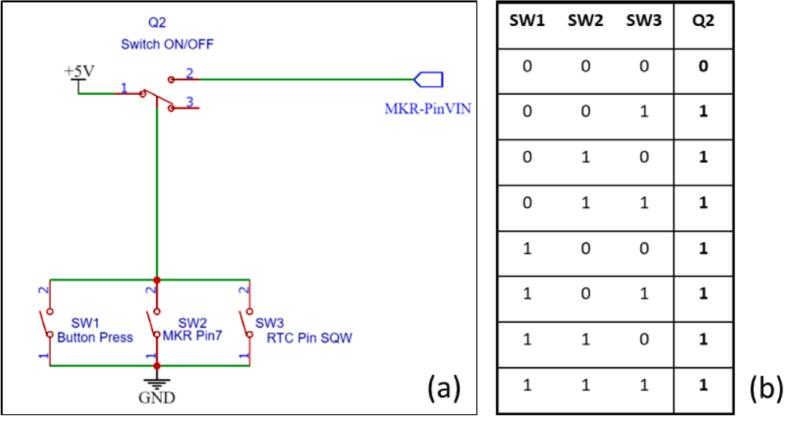
(a) Simplified operating diagram for manual (“SW1” Button Press) and automatic (Pin 7 and Pin SQW taking turns) modes, (b) “or inclusive” truth table, with “0” being the open position and “1” the closed position.
b) During a first use, the SD is initialized by creating the data.txt file on which the data will be written. For every subsequent run, the presence of the SD is verified and indicated to the user via MKR “built-in” LED lighting up for 5sec if detected, or blinking if not detected (Fig. 9).
c) Depending on the sensors connected to the board and the user’s requirements, a series of measurements are performed using subroutines implemented in the Arduino code (Design File 4).
d) After completing the measurements, the microcontroller attempts to connect to the compatible network and transmits the data as a payload. This payload, a hexadecimal value, contains the results of the current measurements. If the connection fails, the data is only stored on the SD card.
e) End of the program:
- The RTC sets an alarm to wake the system after a specified interval (e.g., 5, 10, 15, 20 min, or another user-defined period in the “Sleep_Period” variable);
- The system is then forced to shut down completely to conserve battery power by setting the transistor value to 0 (Fig. 11);
- If shutdown fails and the shutdown code line does not execute, the MKR board will continuously attempt to shut down by executing the command every 5 s in a loop (MKR “built-in” LED flashes to indicate the procedure).
f) The system automatically restarts when the alarm triggers, activating transistor Q2 (Fig. 11) via the RTC. Pin 7 (SWITCH_ON_OFF) takes over, and the alarm is cleared with the code line “DS3231_clear_a2f();”. The program then continues as expected.
g) The system returns to step c), repeating the measurement process as required.

The steps for using the eLogUp! system are as follows:

a) The operator must hold down the SW1 button (Fig. 10) until LED1 on the white board lights up. This indicates that SW1 can be released, signaling the start of the Arduino program. The waiting period corresponds to the time required for the board to initialize, load its bootloader, and execute the initial lines of the program (Fig. 9). Once the program is running, pin 7 (SWITCH_ON_OFF) takes over from the SW1 button (Fig. 11), causing LED1 to stay lit up (Fig. 10).

### 6.2 Main procedures

#### 6.2.1 Procedure for (re)setting the MRK time clock

**Fig. 12:**
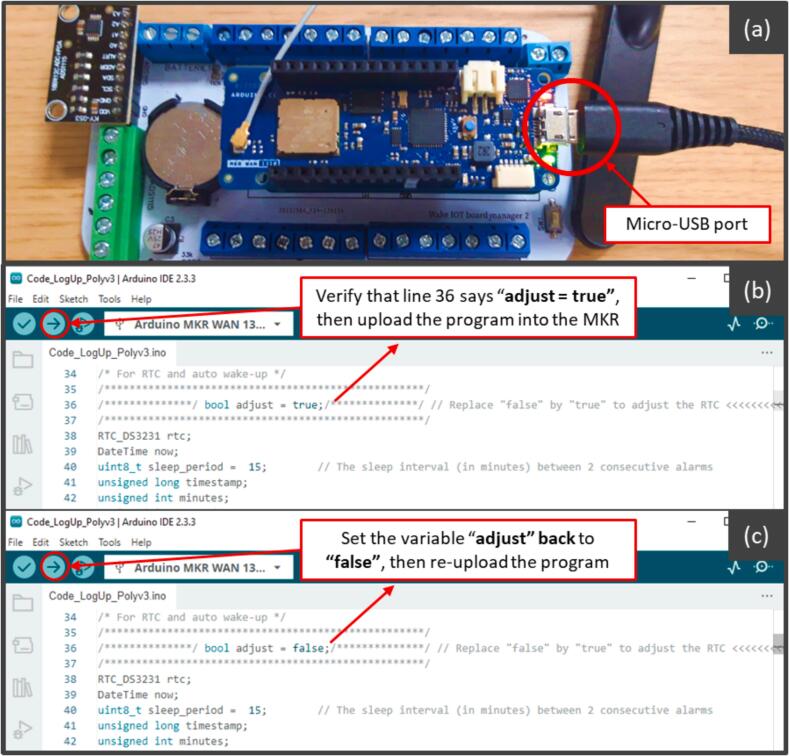
Two-step process to synchronize the RTC with the computer's date and time using the Arduino IDE.

To set or reset the time clock, the user must follow these steps:

1. On the computer (used to upload the program), set the PC time to UTC + 0 and ensure it is synchronized through the internet.

2. Open the Code_eLogUp.ino file in the Arduino IDE (Fig. 12), with the MKR board connected to the computer (either online or offline).

3. In the script, set the variable “adjust” to true.

4. Upload the program to the MKR. The MKR “built-in” LED will blink while the program is running (Fig. 10).

5. Once the program finishes, change the “adjust” variable to false in the script.

6. Re-upload the program with the updated setting.

At this point, the datalogger can be disconnected from the computer, and the SD card should be reinserted. The datalogger will check the timestamp of the latest acquisition on the SD card. Before finalizing the setup, it is advisable to wait for two automatic measurements and check on a computer that the timestamps of the data stored on the SD card are correct.

#### 6.2.2 Procedure for collecting data from the SD card

To collect the data, follow these steps immediately after the last series of measurements to ensure there is enough time to: i) remove the SD card from the MKR MEM SD Shield; ii) copy/paste the data file from the micro SD card to the computer, and iii) reinsert the SD card back into the MKR MEM SD Shield. Wait for one automatic measurement to confirm that the system is functioning properly.

#### 6.2.3 Procedure for data transfer with LoRa WAN network

**Fig. 13:**
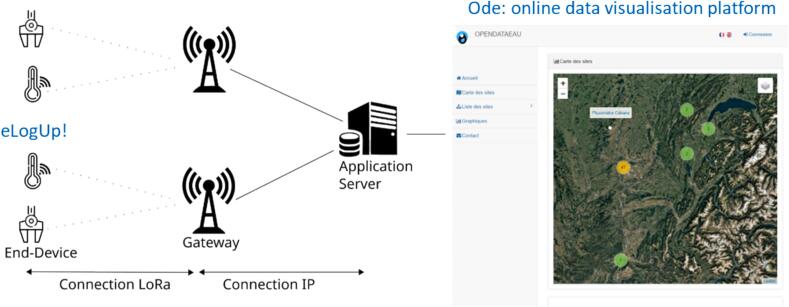
From the field to the office: the eLogUp! system deployed in the field and the Ode online visualization platform (https://opendataeau.org/; modified from https://fr.wikipedia.org/wiki/LoRaWAN).

After each measurement, the encoded data is automatically sent via a LoRa Gateway to an application server (Fig. 13). The LoRa Gateway can be either i) the free TTN operator (The Things Network) if network coverage is sufficient in the study area, or ii) a paid operator. These operators provide tools to decode the received data and transfer it to a storage and visualization server of your choice. Some operators may offer a paid service for this step. Numerous online resources detail the procedures for LoRa data transmission and reception (e.g., https://www.univ-smb.fr/lorawan/wp-content/uploads/2022/01/Book-LoRa-LoRaWAN-and-Internet-of-Things.pdf). In our case, we transmit the data to Ode, a free and open online platform that we developed (https://opendataeau.org/).

#### 6.2.4 Procedure for controlling the battery and solar panel

While in the field, the battery stays mostly charged through the solar panel. To check the battery level, press the Volume switch button, on the regulator (Fig. 7). The number of LEDs lit (1, 2, 3, or 4) will indicate the current battery level. Additionally, the charging status can be monitored using the LEDs on the regulator: i) the “CH” red LED indicates that the solar panel is receiving sunlight; ii) the “OK” green LED signals that the battery is fully charged; iii) if neither LED is lit, it means the solar panel is not receiving sufficient sunlight.

#### 6.2.5 Procedure for switching off the station for a long duration

If the system is not to be used afterward, the flat battery can be simply removed. However, to stop the station without losing the RTC clock, there are two options to prevent the flat battery (CR2032 or CR2016) from draining. The first option is to use the default code (Design File 2), removing the SD card from the MEM Shield will cause the system to shut down automatically after clearing the current alarm. To conserve the battery, the user only needs to remove the SD card between measurements to exit auto-mode. For the second option, if the SD card is not removed, the user should wait for the station to automatically power on (based on the set timestep) and then immediately turn off the power on the LiPo Rider Pro regulator.

## 7 Assessment and characterization

### 7.1 Field applications

Several dozen eLogUp! systems are operational in the field and transmitting various data (water level, turbidity, temperature, humidity, seismic signal; https://opendataeau.org/). We present here only two examples illustrating the eLogUp! system's field operation.

**Fig. 14:**
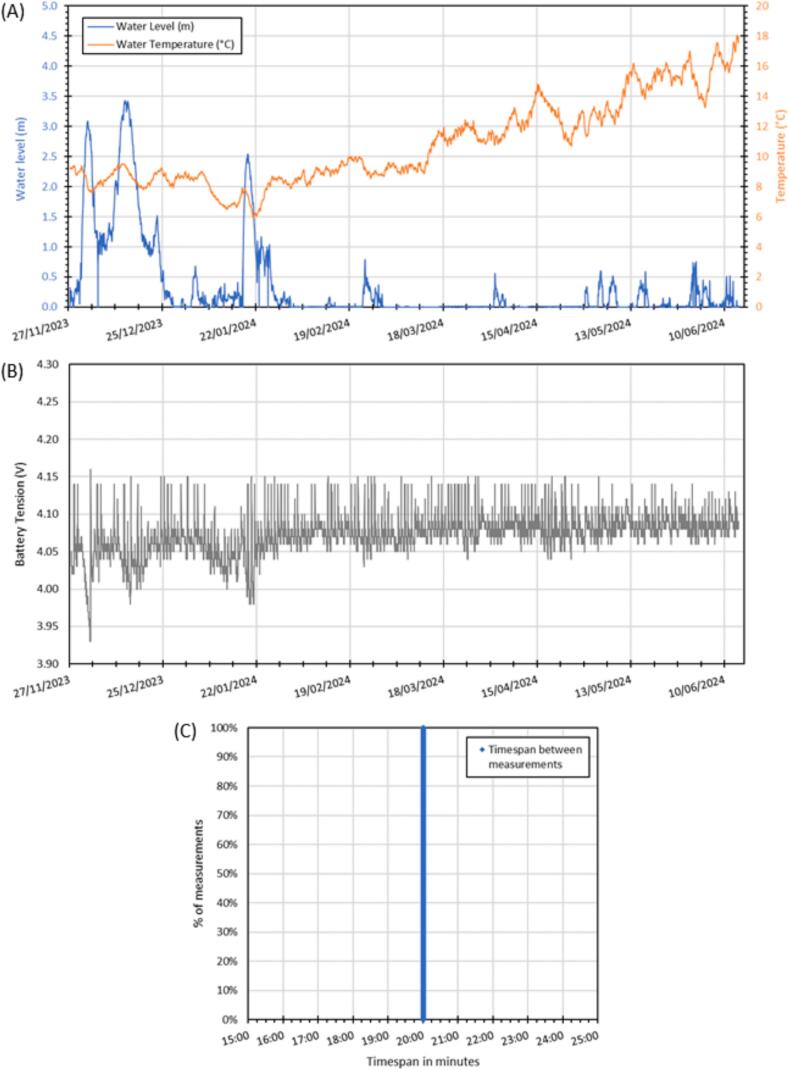
Monitoring of water level and temperature on the Rhône (from 27/11/2023–14/06/2024) with DS18B20 and KIT0139 probes: (A) measurement results; (B) battery voltage; (C) timestep variation statistic distribution.

The first example involves a KIT0139 low-cost piezometer and a DS18B20 temperature sensor [19] to monitor the water level and temperature of the Rhône River in Lyon, France, with measurements recorded every 20 min (Fig. 14). The sensors did not produce any out-of-range measurements (Fig. 14A). The water temperature gradually rises in the spring, while the water level decreases due to fewer rainfall events. To emphasize the effectiveness of the auto wake-up feature, Fig. 14B tracks the battery voltage, demonstrating that the solar panel was sufficient to charge the battery throughout the winter at this study site. The statistical distribution of the time interval shows the RTC's reliability (Fig. 14C). It illustrates accurate timekeeping, down to the nearest second, throughout the entire deployment period. The time intervals between measurements remain consistent with the target 20-minute frequency, highlighting the system's precision in maintaining periodicity.

**Fig. 15:**
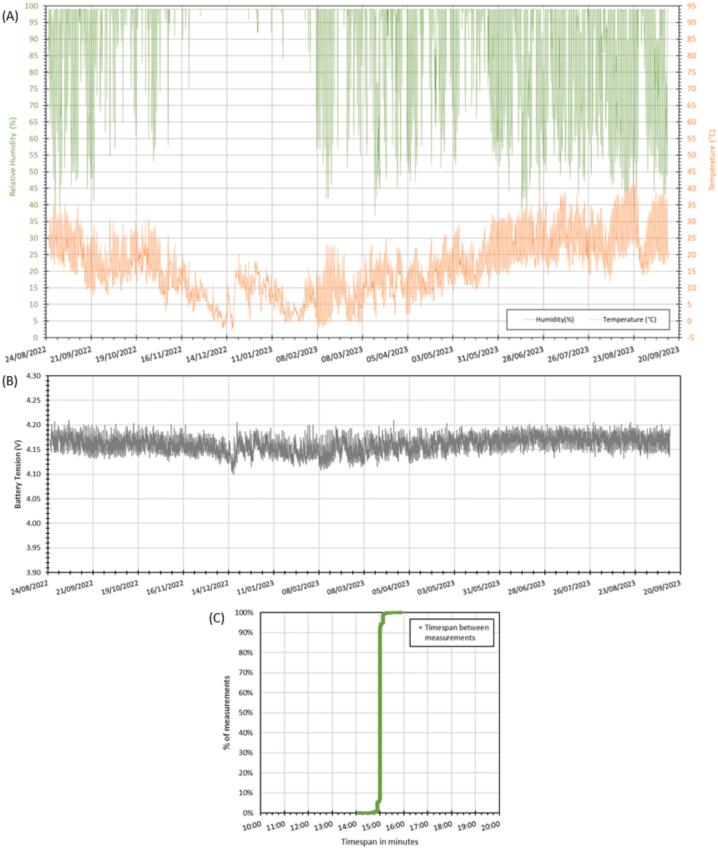
Monitoring relative humidity and air temperature near a tree for more than 1 year (from 08/25/2022 to 09/13/2023) with a DHT22 sensor: (A) measurement results; (B) battery voltage; (C) timestep variation statistics.

A second eLogUp! system was used with a DHT22 sensor to measure air temperature and relative humidity in a tree near an infiltration basin (Bron, France), with a measurement interval of 15 min over a year (Fig. 15). No data loss occurred during the deployment period (Fig. 15A). The battery voltage remained consistently above 4.10 V throughout the deployment (Fig. 15C), confirming the effectiveness of the power system. However, Fig. 15C presents a slight deviation: the cumulative percentage of measurement points compared to the targeted timestep does not demonstrate precision to the second. Instead, the system appears to wake up at 15 ± 1-minute (based on the RTC’s time reference). This issue is primarily linked to the LoRa connection, which occurs before the measurement series and the RTC time request, causing some variability in the timestamp. To address this, the Arduino code for eLogUp! has been modified to ensure the system queries the network only after each measurement is completed (Design File 2).

As noted in previous sections, the DS3231 RTC module is accurate to the second within its own time reference frame. However, during field tests, a notable observation was made regarding the module's tendency to either lag behind or lead ahead of real time. This drift appears to be influenced by factors such as battery voltage and ambient temperature. According to the manufacturer, the drift is estimated to be approximately 1 min per year [23]. While this drift results in a loss of precision in the actual timestamp, the interval between two consecutive measurements remains consistent over time. The advantage of using the alarm function outweighs this drift. However, simple solutions exist to address the time discrepancy, such as manually synchronizing the RTC during field visits or using an additional GPS module to retrieve the real-time timestamp via the internet and synchronizing the RTC accordingly. When using the MKR 1500, the RTC can be updated each time the system connects to the GSM network, ensuring that the RTC remains synchronized, thereby minimizing any discrepancy.

### 7.2 Evaluation of the energy consumption of the eLogUp! system

To assess the energy consumption of the eLogUp! system, battery life tests were conducted under controlled laboratory conditions. The eLogUp! monitoring system was tested with different sensors, including a JST-SR04T ultrasonic distance sensor and a DS18B20 temperature probe (with its subroutine detailed in Design File 2). The “Sleep_Period” variable in the Arduino code was set to 15 min to align with LoRaWAN recommendations. The laboratory temperature remained constant at approximately 20 °C. In this experiment, the solar panel was removed to simulate a malfunction or an extended period without sunlight.

Six tests were performed (labeled from a to f; Table 4), monitoring the voltage drop over time of the same fully recharged 4000 mAh LiPo battery, from 4.10 V until the MKR1310 stopped functioning. The eLogUp! PCB features an auto-wake-up mode, enabling the system to power down completely between acquisitions (test e). To evaluate its efficiency, this mode was compared with the “delay” function (test a) and the microcontroller’s built-in “sleep” and “deep sleep” modes (tests b and c, respectively). Additionally, we tested powering the MKR 1310 without auto-wake-up, connecting it directly via the JST port while using the “deep sleep” mode (test d). Lastly, we assessed the power consumption of the Lipo Rider regulator (test f) within the overall energy usage of the eLogUp! system. Given the voltages involved, there is no issue not using any regulator, as MKR boards contains its own voltage regulator (https://docs.arduino.cc/tutorials/mkr-wifi-1010/powering-with-batteries/). With a solar panel, we however recommend using the DFR0264 module to safely charge the LiPo battery (https://wiki.dfrobot.com/Solar_Charger_SKU_DFR0264).

Design files are available at the repository: https://osf.io/96zv2/?view_only=5a670b5f49634e0cb3b69654044ec012.

**Fig. 16:**
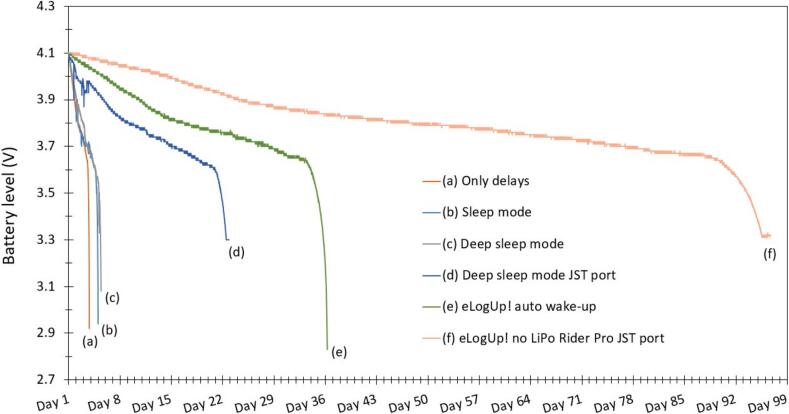
Full battery discharge over time according 6 tests (Tab. 3): (a) only the “delay” function; (b) “sleep” mode; (c) “deepSleep” mode; (d) “deepSleep” mode by powering the MKR1310 through the JST port; (e) eLogUp!’s auto wake-up system with the LiPo Rider Pro; (f) eLogUp!’s auto wake-up system by powering through the JST port, without LiPo Rider Pro.

**Fig. 17:**
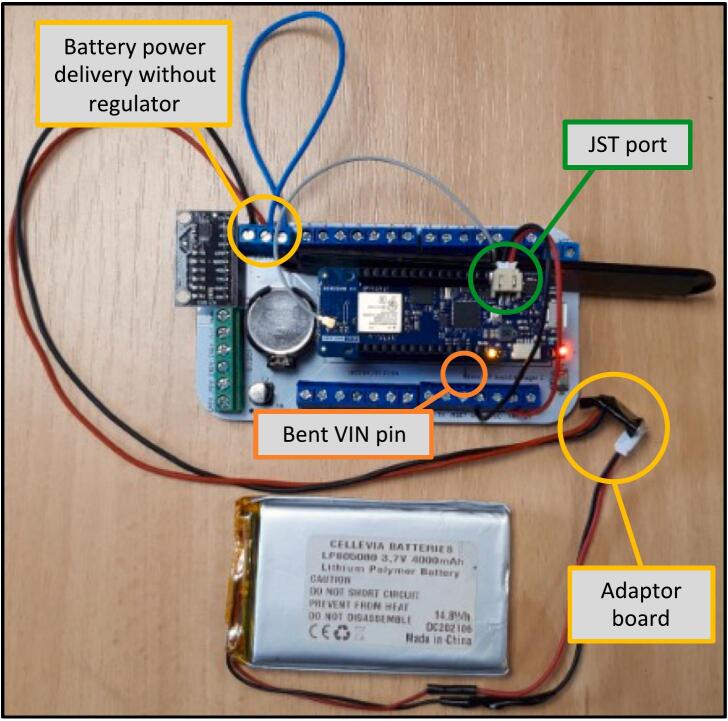
Maximum optimization of battery life with JST port and without LiPo Rider Pro (test f).

A total of 295 measurements were recorded using the "delay" function without any power-saving 510 optimizations (test a), leading to a runtime of less than 3 days (Fig. 16). This short duration was expected 511 since all LEDs remained constantly ON, significantly depleting the battery. For tests b and c, the “sleep” and “deepSleep” modes were enabled with either the function 520 LowPower.sleep() or LowPower.deepSleep(). When in sleep mode, the power LED remains dimly lit, 43/46 contributing to energy savings. Additionally, the SWITCH_ON_OFF command 521 was set to LOW throughout 522 the program execution, ensuring that LED1 on the eLogUp! PCB remained OFF (Fig. 10), as the LED is not 523 used when powering the MKR via the JST port. As a result, in test b (“sleep” mode), the system operated 524 for 4 days. In test c (“deepSleep” mode), the runtime extended to 5 days, showing only a slight 525 improvement over test b (Fig. 16). 526 In test d, where the system was powered solely via the JST port and operated in deep sleep mode, 527 the runtime extended to over 23 days. This significant increase in battery life was due to the internal 528 voltage management of the Arduino board and the absence of the LiPo Rider Pro, which otherwise 529 contributes to power consumption. As shown in Fig. 16, the battery voltage stabilizes at 3.3 V during the 530 final measurements, which corresponds to the nominal operating voltage of the MKR when powered 531 directly through the JST port (Fig. 17). 532 533

## 8 Conclusion

The eLogUp! system represents a highly versatile, accessible, and scalable solution for both research 548 oriented and operational field projects requiring a reliable, cost-effective, and fully open-source 549 environmental datalogging platform. Designed with adaptability in mind, it supports a wide range of 550 sensor types, making it suitable for diverse applications – from environmental monitoring and hydrology, 551 air pollution to agriculture and infrastructure assessment. This flexibility is enabled by the Arduino 552 ecosystem, which continually expands the system’s capabilities through community-developed libraries 553 and support for various communication protocols, including SDI-12, I2C, UART. As a result, eLogUp! not 554 only lowers the barrier to entry for complex data acquisition tasks, but also empowers users to customize 555 and scale their deployments according to specific project needs and constraints. 45/46 Looking ahead, upcoming PCB upgrades are set to significantly improve 556 usability and integration. 557 These include repositioning the “VCC” and “GND” terminal blocks for more intuitive wiring, integrating a 558 dual terminal block for external activation prior to scheduled alarms, and embedding JST ports to simplify 559 power connections directly from batteries and solar panels – eliminating the need for additional regulator 560 components. 561 The eLogUp! system continues to evolve to meet field requirements, while maintaining its core 562 principles of openness, flexibility, and cost-effectiveness. Ongoing improvements aim to enhance the 563 platform’s performance, the environmental cost and simplify its deployment, thereby expanding its 564 potential use in areas such as environmental monitoring, scientific observation, and citizen science.

## Declaration of generative AI and AI-assisted technologies in the writing process

During the preparation of this work the authors used ChatGPT in order to improve language and editing. After using this tool/service, the authors reviewed and edited the content as needed and take full responsibility for the content of the publication.

## CRediT authorship contribution statement

**Franck Perret:** Writing – original draft, Visualization, Validation, Supervision, Software, Methodology, Investigation, Formal analysis, Conceptualization. **Ilane Cherif:** Writing – review & editing, Writing – original draft, Visualization, Validation, Software, Resources, Methodology, Investigation, Formal analysis. **Frédéric Cherqui:** Writing – review & editing, Validation, Supervision, Software, Resources, Project administration, Methodology, Investigation, Funding acquisition, Formal analysis, Data curation, Conceptualization. **Nicolas Walcker:** Writing – review & editing, Validation, Software, Resources, Methodology, Investigation, Data curation, Conceptualization. **Adrien Barra:** Writing – review & editing, Validation. **Bastien Bourjaillat:** Visualization, Validation, Supervision, Software, Resources, Methodology, Formal analysis. **Laëtitia Bacot:** Project administration, Funding acquisition, Conceptualization. **Oldrich Navratil:** Writing – review & editing, Writing – original draft, Supervision, Resources, Project administration, Methodology, Investigation, Funding acquisition, Formal analysis, Data curation, Conceptualization.

## Declaration of competing interest

The authors declare that they have no known competing financial interests or personal relationships that could have appeared to influence the work reported in this paper.
